# Less abundant bacterial groups are more affected than the most abundant groups in composted tannery sludge-treated soil

**DOI:** 10.1038/s41598-018-30292-1

**Published:** 2018-08-06

**Authors:** Ana Roberta Lima Miranda, Jadson Emanuel Lopes Antunes, Fabio Fernando de Araujo, Vania Maria Maciel Melo, Walderly Melgaco Bezerra, Paul J. Van den Brink, Ademir Sergio Ferreira de Araujo

**Affiliations:** 1 Federal University of Piauí, Department of Agricultural Engineering and Soil Science, Teresina, 64049-550 Brazil; 2 Universidade do Oeste Paulista, Presidente Prudente, Brazil; 3 Federal University of Ceará, Lembiotech, Fortaleza, Brazil; 4 Wageningen University, Aquatic Ecology and Water Quality Management Group, Wageningen, P.O. Box 47, 6700 AA The Netherlands; 5 Wageningen Environmental Research (Alterra), Wageningen, P.O. Box 47, 6700 AA The Netherlands

## Abstract

The application of composted tannery sludge (CTS) has promoted shifts in soil chemical properties and, therefore, can affect the soil bacterial community. This study assessed the effect of the CTS on the soil bacterial community over time. The CTS was applied at five rates (0, 2.5, 5, 10 and 20 t/ha), and the bacterial community was evaluated for 180 days. The principal curve response (PRC) analysis showed that the most abundant phyla were not influenced by the CTS rates over time, while the analysis of the bacterial community showed that some of the less abundant phyla were influenced by the CTS rates. Similarly, the PRC analysis for the bacterial classes showed the significant effect of the CTS rates. The redundancy analyses for the bacterial phyla and classes showed the relationship between the significant chemical properties and the bacterial community of the soil after the CTS amendment over time. Therefore, there was a shift in the bacterial community over time with the application of the composted tannery sludge. Our study has shown that the less abundant bacterial groups were more influenced by the CTS than the most abundant bacterial groups and that these bacterial groups were driven by soil chemical properties, primarily chromium (Cr) and the soil pH.

## Introduction

The generation of solid wastes is increasing worldwide, and therefore, it becomes necessary to find suitable methods for waste disposal in the environment. Unfortunately, in some regions, solid wastes are disposed in the environment without treatment and prevention. This practice has increased the accumulation of pollutants and thus promoted environmental pollution.

Specifically, tannery industries have released annually high amounts of tannery sludge (TS), a type of solid waste that contains large amounts of chromium (Cr), salts, carbonates, and hydroxides^1^. Although it is well-known that TS transfers these chemical elements, this has not precluded its use as a soil conditioner in agriculture^2,3^. Consequently, this practice has already promoted the accumulation of Cr and the salinization of soils in some regions, affecting the soil microbial properties and plant growth^4^.

Currently, new methods to detoxify TS before its use have been proposed, and composting has been reported to be an efficient method^1^. The use of composted tannery sludge (CTS) has improved the soil organic matter content and concentrations of plant nutrients, such as P, K, and Ca^1,5^. However, the permanent amendment of CTS has promoted the accumulation of Cr and can cause soil pollution^5^. Therefore, the application of the CTS may affect the soil properties, primarily the microbial communities that are driven by soil properties.

As expected, previous studies have found a significant influence of the CTS on the chemical properties of soil^5^, and these changes have affected the soil microbial biomass and enzyme activities^1^. However, it is unclear how the CTS affects the bacterial community over time, i.e., what the time-dependent effects of CTS are on the bacterial community and structure. Although previous studies have assessed the effect of the TS on the bacterial community^6,7^, the effect of the CTS on bacterial community remains unclear.

The bacterial community plays important roles in the soil ecosystem and is responsible for processes such as organic matter decomposition, mineralization, and plant growth promotion^8^. However, in the soil ecosystem, the bacterial community is influenced by several biotic and abiotic factors, such as chemical elements in the soil. Therefore, the evaluation of the effect of the CTS amendments, and consequently the changes in the soil chemical properties, on the soil bacterial community is important to determine before its use. Currently, the primary method to assess the soil bacterial community is next-generation high-throughput sequencing^9^, which is based on sequencing the 16S rRNA genes recovered from the environment.

However, this method was not previously used to assess the time-dependent effects of the CTS on the bacterial community. To fill this research gap, we have addressed two primary hypotheses in this study: a) the dynamics of the soil bacterial community are affected by the CTS in the short-term, and b) the changes in the chemical properties of the soil drive changes in the bacterial community.

## Results

### Chemical properties

**Figure 1:**
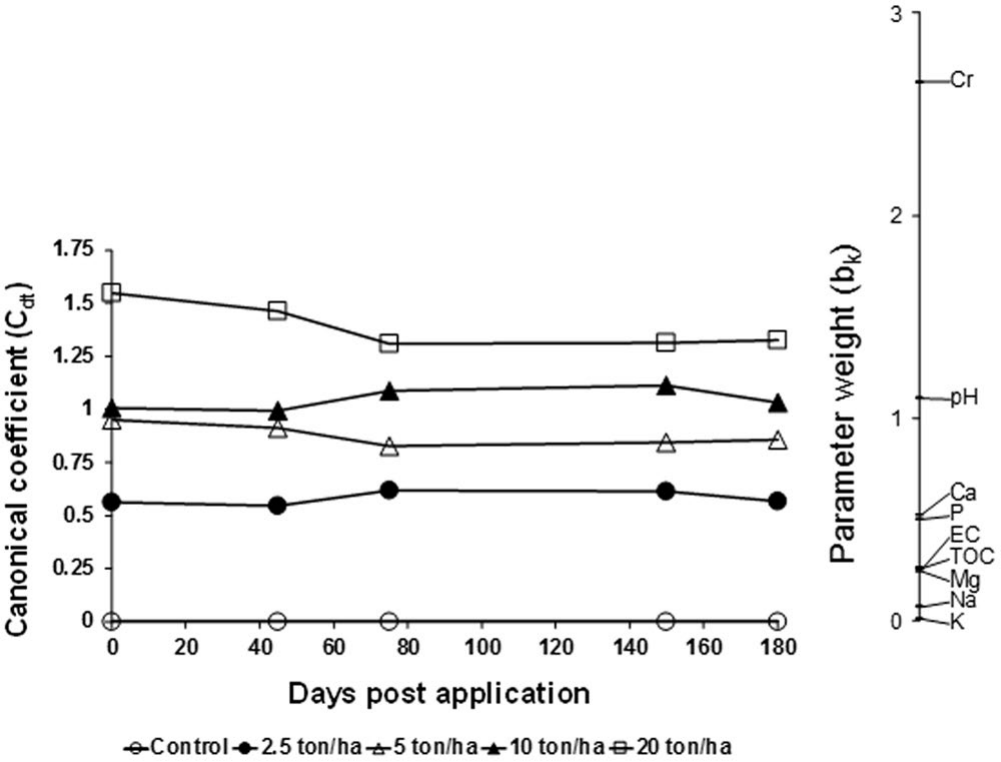
Principal response curve (PRC) diagram of physico-chemical data set indicating the effects of the composted tannery sludge (CTS) into the soil. Of all variance, 7% could be attributed to sampling date; this is displayed on the horizontal axis. 85% percent of all variance could be attributed to treatment. Of this variance, 96% is displayed on the vertical axis. The lines represent the course of the treatment levels in time. The parameter weight (b_k_) can be interpreted as the affinity of the physico-chemical parameters with the PRC. The Monte Carlo permutation test indicated that a significant part of the variance explained by treatment is displayed in the diagram (p ≤ 0.001). The second PRC was not significant. Cr – chromium; pH – soil pH; Ca – calcium; P – phosphorus; EC –electric conductivity; TOC - total organic carbon; Mg – magnesium; Na – sodium; K – potassium.

Principal response curve (PRC) analysis of the chemical properties showed that the CTS rates explained 85% of the total variance and indicated that the values of most of the chemical variables increased in all CTS treatments (no observed effect concentrations; NOEC < 2.5 t/ha; Table S1). In contrast, the chemical properties did not change over time (Fig. 1). Cr and the soil pH were the primary chemical variables influenced by the CTS amendment, showing a weight (*b*_*k*_) higher than 1.0, followed by Ca, P, electric conductivity (EC) and total organic C (TOC) with a weight (*b*_*k*_) higher than 0.25.

### Effects on bacterial phyla

**Figure 2:**
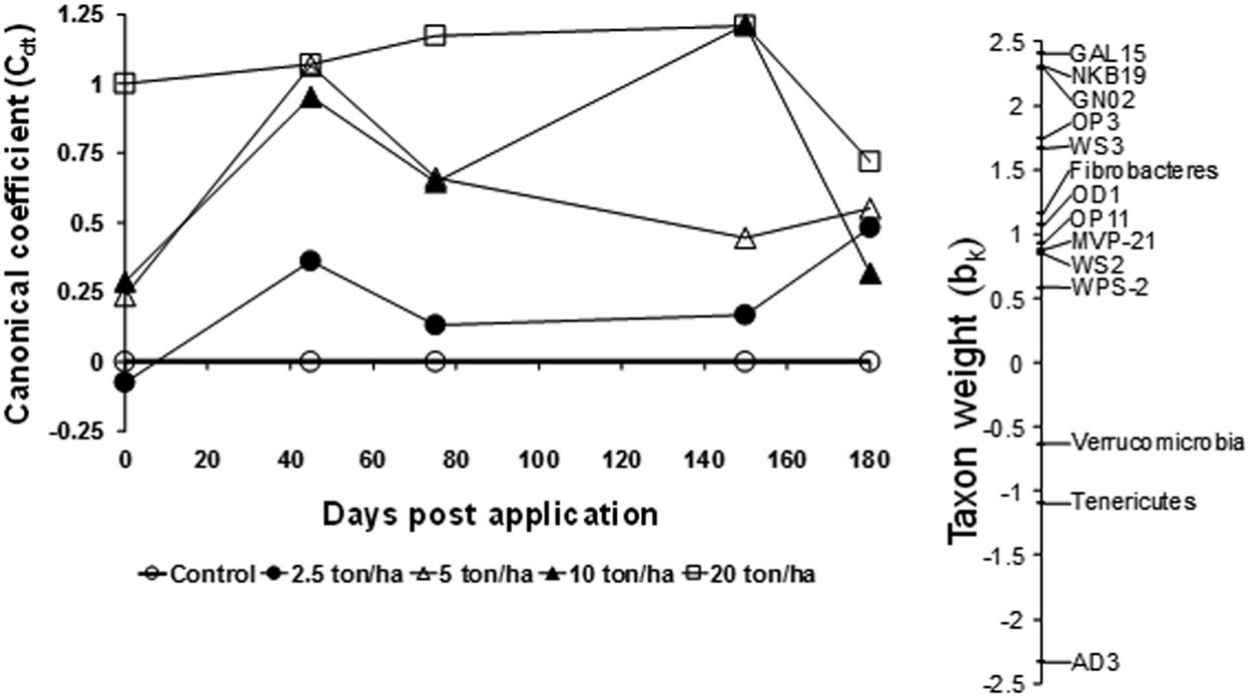
Principal response curve (PRC) diagram of OTU data at Phyla level data set indicating the effects of the composted tannery sludge (CTS) into the soil. Of all variance, 18% could be attributed to sampling date; this is displayed on the horizontal axis. 28% percent of all variance could be attributed to treatment. Of this variance, 36% is displayed on the vertical axis. The lines represent the course of the treatment levels in time. The taxon weight (b_k_) can be interpreted as the affinity of the OTU’s with the PRC. The Monte Carlo permutation test indicated that a significant part of the variance explained by treatment is displayed in the diagram (p ≤ 0.001).

Our study found 16,494 bacterial operational taxonomic units (OTUs) affiliated with 35 phyla, 104 class, and 209 genera. The most abundant bacterial phyla (percentages of OTUs above 1%) were Acidobacteria, Actinobacteria, Bacteroidetes, Chloroflexi, Firmicutes, Gemmatimonadetes, Nitrospirae, Proteobacteria, Planctomycetes and Verrumicrobia (Fig. S1). However, these most abundant phyla were not influenced by the CTS rates over time (Table S1). In contrast, the PRC analysis of the bacterial community showed that some of the less abundant phyla were influenced by the CTS rates (Fig. 2). The analysis for the bacterial phyla showed that 35% and 11% of all variance was explained by time and the CTS rates, respectively. The canonical coefficient (*C*_*dt*_) showed a difference between amended and unamended soils over time, with a greater difference at the highest CTS rate (NOEC = 2.5 t/ha; Table S1). The CTS rates negatively affected the phyla AD3, Tenericutes, and Verrumicrobia with weights (*b*_*k*_) below −0.5. On the other hand, the phyla GAL15, NKB19, GN02, OP3, and WS3 were positively influenced by the CTS rates with weights (*b*_*k*_) above 1.5.

### Effect on bacterial classes

**Figure 3:**
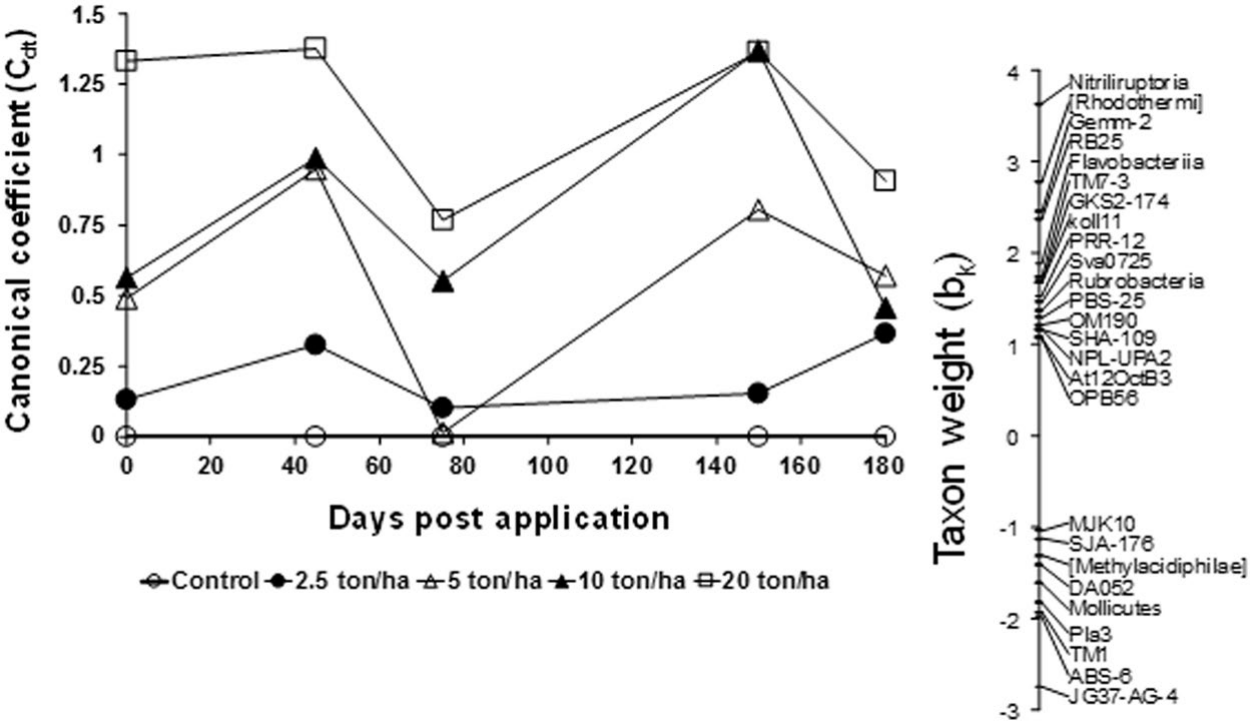
Principal response curve (PRC) diagram of OTU data at Class level data set indicating the effects of the composted tannery sludge (CTS) into the soil. Of all variance, 20% could be attributed to sampling date; this is displayed on the horizontal axis. 30% percent of all variance could be attributed to treatment. Of this variance, 32% is displayed on the vertical axis. The lines represent the course of the treatment levels in time. The taxon weight (b_k_) can be interpreted as the affinity of the OTU’s with the PRC. The Monte Carlo permutation test indicated that a significant part of the variance explained by treatment is displayed in the diagram (p ≤ 0.001).

Similarly, the PRC analysis for the bacterial classes showed a significant effect of the CTS rates (Fig. 3). The results showed different patterns between the bacterial classes in the CTS-treated soils and the unamended soil, except for the lowest and highest CTS rates in different periods of sampling (NOEC = 2.5 t/ha at 45 and 150 days of sampling and NOEC > 20 t/ha at 75 and 180 days of sampling; Table S1). The classes were positively or negatively influenced by the CTS rates over time. In the analysis for the classes, we only considered weight values (*b*_*k*_) greater or lower than 1.0 and −1.0, respectively. Interestingly, the PRC for the classes also showed an increase in the *C*_*dt*_ at 45 and 150 days, while it decreased at 75 and 180 days. For example, the class *Nitriliruptoria* (NOEC = 2.5 ton/ha based on an increase at 45 and 150 days of sampling and NOEC = 10 ton/ha also based on an increase at 75 and 180 days of sampling) was positively influenced by the CTS, while the class *JG37-AG-4* (NOEC = 5 ton/ha based on a decrease at 45 and 150 days of sampling and NOEC > 20 t/ha at 75 and 180 days of sampling) was negatively affected by the CTS amendment over time.

### Multivariate responses

**Figure 4:**
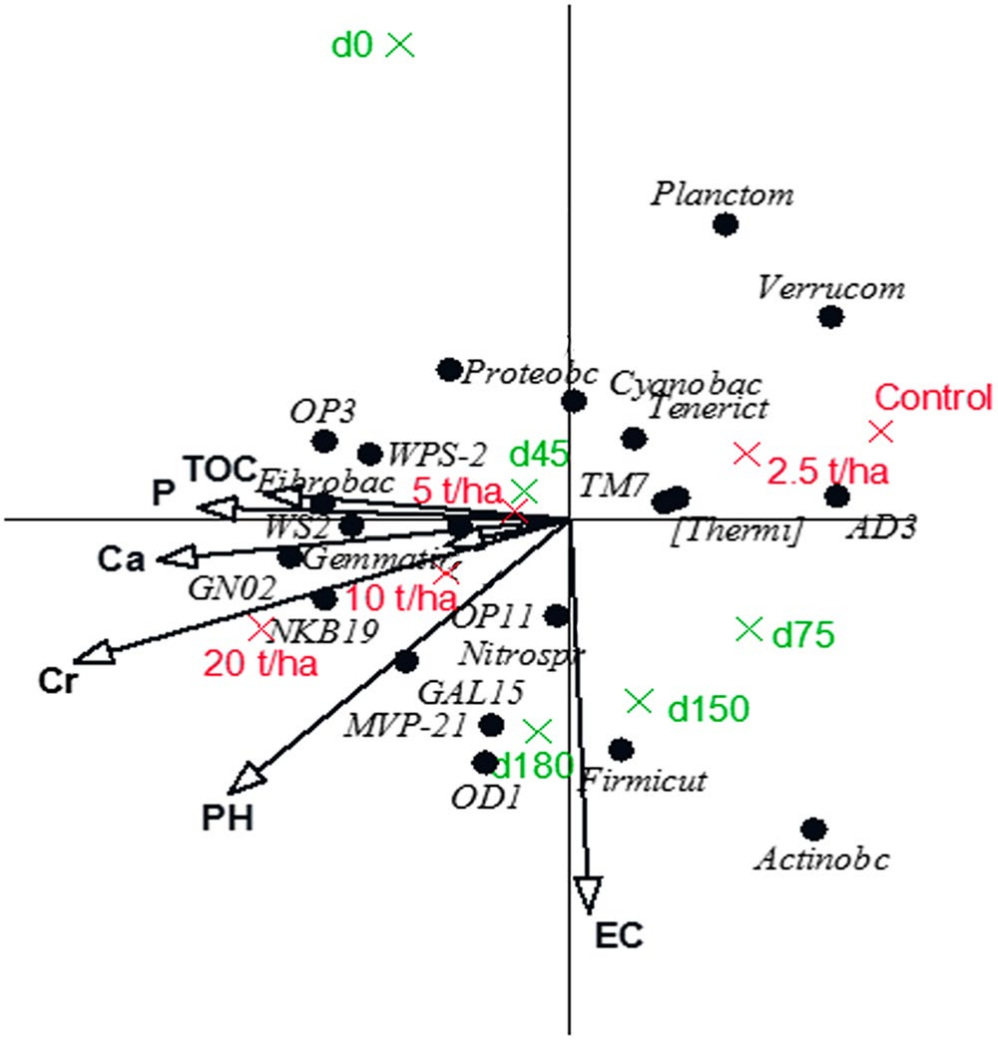
Redundancy analysis diagram (RDA) of correlations between significant physico-chemical proprieties and OUT’s at the Phyla level. Physico-chemical properties were introduced as explanatory variables and explained 27% of the total variation of which 37% is displayed on the horizontal axis and another 30% on the vertical one. The variables treatment and sampling date were introduced as supplementary environmental variables. Rates of CTS (2.5, 5, 10 and 20 t/ha^−1^); Time of sampling in days (0, 45, 75, 150 and 180); Total organic carbon – TOC; P – phosphorus; Ca – calcium; Cr – Chromium; pH – soil pH; EC – electric conductivity.

**Figure 5:**
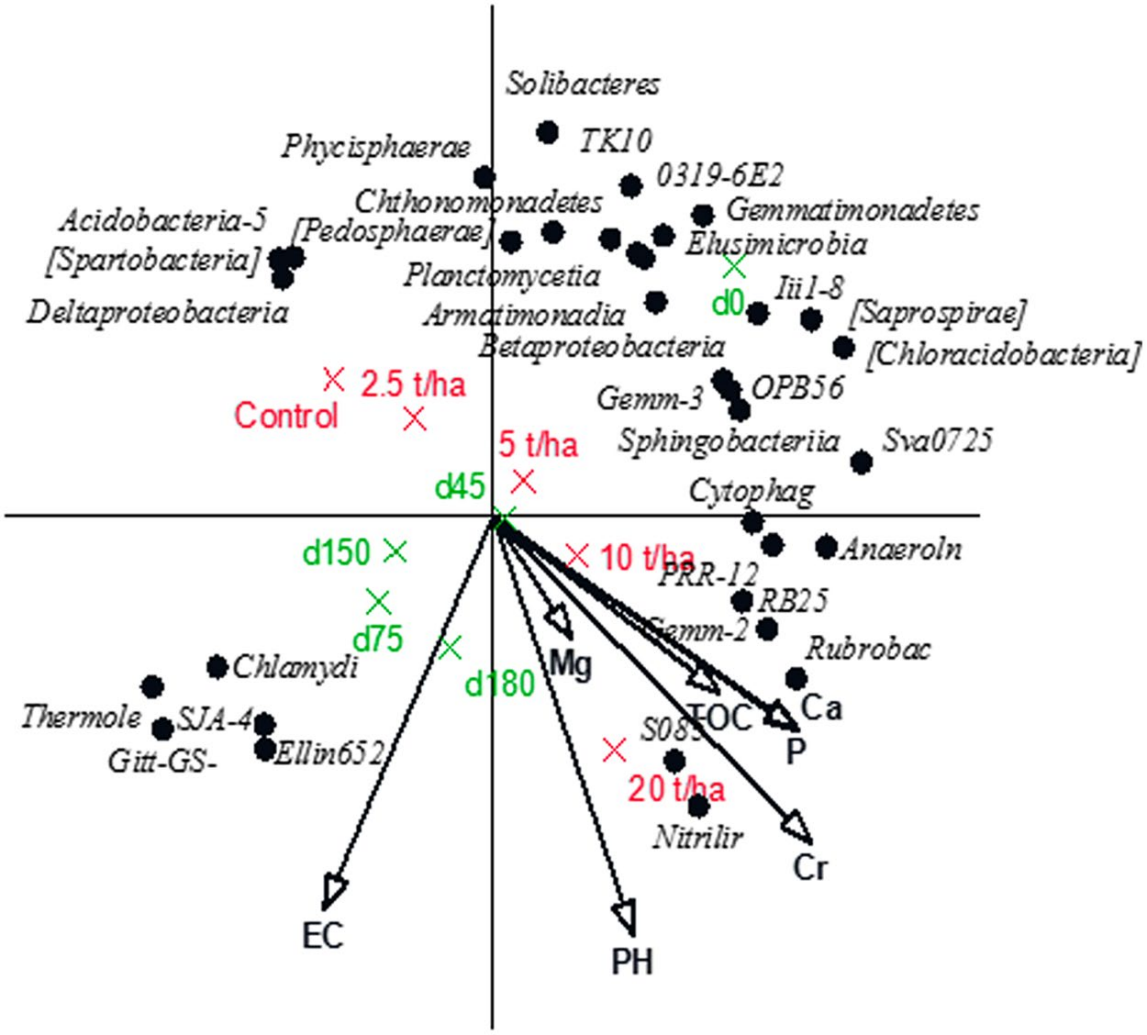
Redundancy analysis diagram (RDA) of correlations between significant physico-chemical proprieties and OUT’s at the Class level. Physico-chemical properties were introduced as explanatory variables and explained 25% of the total variation of which 47% is displayed on the horizontal axis and another 24% on the vertical one. The variables treatment and sampling date were introduced as supplementary environmental variables. Rates of CTS (2.5, 5, 10 and 20 t/ha^−1^); Time of sampling in days (0, 45, 75, 150 and 180); Total organic carbon – TOC; P – phosphorus; Ca – calcium; Cr – Chromium; pH – soil pH; EC –electric conductivity. For clarity only the 34 classes of which more than 30% of its variation is displayed on both axes are shown.

The redundancy analyses (RDA) for the bacterial phyla and classes showed the relationship between the significant chemical properties and the bacterial community of the soil after the CTS amendment over time (Figs 4 and 5, respectively). These analyses showed that 27% and 25% of the variation for the bacterial phyla and classes, respectively, were explained by the chemical properties influenced by the CTS amendment. For both the phyla and the classes, the results showed significant chemical variables, i.e., TOC, P, Ca, Cr and pH, clustered with the highest CTS rates (10 and 20 t/ha). For the phyla, these chemical variables and the highest CTS rates clustered with Fibrobacteres, WS2, Gemmatimonadetes, GN02, NKB19, OP11, Nitrospira, GAL15, MVP-21, and OD1. In contrast, we found that the phyla Planctomicetes, Verrucromicrobia, Cyanobacteria, Tenericutes, TM7, [Thermi] and AD3 had a negative relationship with the chemical properties, thus clustering with the lowest CTS rate (2.5 t/ha) and the unamended soil. For classes, these variables and the highest CTS rates clustered with *Nitriliruptoria, S085, Rubrobacteria, RB25, PRR12, Anaerolinea*, and *Gemm-2*.

The RDA also showed the pattern of the bacterial community over time. From 0 days (without rhizospheric effects) to 180 days (rhizospheric effects with maize and cowpea), there was a shift in both the phyla and classes. However, the bacterial communities associated with 45 days (the flowering of maize) were separated from the bacterial groups clustered with 75 days (the senescence of maize). In contrast, the bacterial communities associated with cowpea were more similar at 150 days (the flowering of cowpea) and 180 days (the senescence of cowpea).

## Discussion

As expected, the application of the CTS strongly increased some chemical variables, such as Cr, pH, Ca, P, EC, and TOC. This confirms that the chemical characteristics of the CTS (Table 1) influenced the increases in these variables, primarily Cr and the pH. However, there were no changes in the chemical properties over time, probably because the CTS had only been applied at the beginning of the experiment without additional amendments for 180 days.

**Table 1:** Chemical attributes of the CTS used in the experiment.

pH	Moisture	TOC	N	P	K	Ca	Mg	Na	S	Cu	Ni	Cd	Cr	Pb
H_2_O	%	----------------------g kg^−1^---------------------								----------mg kg^−1^----------				
7.5	68	201	15	4.9	2.9	121	7.2	49.1	10	16	23	1.9	1,943	40
MLP^*^	—	—	—	—	—	—	—	—	—	200	70	3	150	180

^*^Maximum limit permitted by Brazilian regulation^48^.

Cr was the primary chemical element that increased most significantly, and its increase with the application of CTS can be deleterious to the soil and the plants^10,11^. Although the application of the CTS promoted the increase in the organic matter content in the soil^5^ and could decrease Cr availability, the presence of this element could change the structure of the microbial community^7^.

Acidobacteria and Actinobacteria were the most abundant phyla found in our study, and this is consistent with previous studies that found that these phyla are the most abundant in unpolluted soils^12^ and soil polluted with Cr^13^. Additionally, we found that the phyla Bacteroidetes, Chloroflexi, and Firmicutes were more abundant than Proteobacteria, which could suggest that the application of the CTS has influenced these groups to be more abundant than Proteobacteria, probably because Bacteroidetes, Chloroflexi, and Firmicutes are more resistant to the presence of metal and chemical modifications in the soil.

However, these most abundant phyla were not influenced by either the CTS rates or time. Interestingly, our results showed that the less abundant phyla were affected by the CTS rates and time. In particular, the phyla AD3, Tenericutes and Verrumicrobia were negatively influenced by the CTS over time, which can be explained by the strict relationship between these organisms with the pH, organic carbon, nitrogen and electric conductivity^14^, showing greater abundance in sites with low concentrations of C and N and salinity, although AD3 showed higher abundance in soil with a higher pH^15^.

Alternatively, the phyla GAL15, NKB19, GN02, OP3, and WS3 were positively influenced by the CTS due to the positive correlation with salinity and high soil pH. This can be explained by the well-known adaptation of these organisms to fertile soils with low acidity, as well as their presence in residual treatment waters^14,16–18^. Previous studies have shown that the bacterial community is influenced by the application of industrial wastes, such as tannery sludge, that change the chemical properties of soil, primarily the soil pH and salinity^19^, as well as the available Ca, Mg, and Mn^20^. Therefore, these changes in the soil chemical properties could distribute the bacterial phyla at local scales.

Although CTS is considered to be rich in chemical compounds, such as Na, Ca, Mg and K, these parameters demonstrated in our study had a relatively low weight within the primary PRC. This can be explained by the absorption, adsorption, and leaching of the salts, promoting the reduction of the electrical conductivity in the soil^21^. Even given the importance of the pH and temperature parameters for the microbiota, salinity is considered to be a very important factor for the microbial environment, since it more directly influences the community structure than any other chemical factor in extreme values^22^.

Several bacterial classes were influenced by the application of the CTS over time showing negative and positive effects. It confirms our first hypothesis (a) that the bacterial community would change with the application of the CTS over time. Alternatively, the different CTS rates varied in their influence on the properties of the soil, as discussed above, and consequently affected the bacterial groups.

Alternatively, the presence of different plant species cropped over time modified the soil environment, primarily the rhizosphere that influenced the bacterial groups. Specifically, the bacterial classes showed a different pattern according to periods of 45 and 150 days and 75 and 180 days. It could be related to the presence of the rhizosphere of different plants and their activities. The periods 45 and 150 days corresponded to the flowering of maize and cowpea, while 75 and 180 days corresponded to the senescence of both crops. These results showed a strong relationship between the flowering and senescence periods of these crops and the activity of the rhizosphere and consequently on the bacterial community.

The rhizosphere strongly influences the microbial communities directly, such as increasing enzyme activities (amylase, urease, cellulase and protease), and indirectly, as an increase in the soil chemical attributes^23^. Additionally, the rhizospheric effect varies according to the plant species and the stage of development^23,24^. Therefore, the pattern of increase in the abundance of bacterial classes during the flowering stages could be explained by higher activity in the rhizosphere, favouring the bacterial community^25^. In contrast, during senescence, the plant decreases its absorption of nutrients and thus decreases the activity in the rhizosphere.

Although the PRC diagram at the bacterial class level does not show a significant difference between the treatments (Fig. 3), it is possible to observe a distribution of the classes with a higher or lower sensitivity to the CTS. As expected, some of these classes belong to those with higher or lower sensitivity phyla found in the PRC for phyla. Therefore, we found distinct bacterial groups in the rhizosphere of maize during flowering (Acidobacteria-5, [Spartobacteria], Deltaproteobacteria and Phycisphaerae) and plant senescence (Chlamydiia, Thermoleophilia, SJA-4, Giit-GS-136 and Ellin6529). In contrast, we did not find distinct bacterial groups during the flowering and senescence of cowpea. This suggests that cowpea can maintain the same bacterial community, while maize promotes a shift in bacterial groups. Therefore, different plant species demonstrate distinct rhizospheric effects that then affect the bacterial community^26^. Additionally, the quality and quantity of the roots exudates can be determined by the plant species^24^ and the stages of growth that release specific substrates and potentially antimicrobial compounds selecting specific microbial groups in the rhizosphere^23,27^.

The chemical properties (pH, Cr, P, TOC, Ca, Mg and EC) showed a direct influence on the classes Nitriliruptoria and Rubrobacteria (Actinobacteria), Anaerolinea and S085 (Chloroflexi), RB25 (Acidobacteria), PRR12 (WS3) and Gemm-2 (Gemmatimonadetes), specifically, in the highest rates of the CTS. It is known that these organisms are sensitive to the presence of organic matter in the environment, resulting in variable responses to the pH and availability of nutrients^12,20^. Additionally, Cr strongly influenced the bacterial classes. Metal stress affects sensitive species^28^ and decreases their ability to compete, resulting in an increase in the abundance of metal-resistant species that can adapt to stress and fill the empty niches in order to maintain ecological stability (functional redundancy)^29^. The chemical characteristics of the soil directly interfere with the behaviour and distribution of the microorganisms, both with respect to the chemical composition and the time of exposure to these factors^19,30^.

Consistent with the second hypothesis (b), the bacterial community is influenced by the change in chemical properties after the application of the CTS in the soil. Our study has shown that the application of CTS promoted an increase in specific groups that were metabolically adapted to the disturbed environment. It is important to select bacteria for bioremediation purposes. For example, Nitriliruptoria is a bacterium that is adapted to high pH values and produces enzymes able to metabolize nitriles and offers great promise for environmental biotechnology^31^. Similarly, Rubrobacteria is found in polluted environments and could be useful for bioremediation^32^. Our study also found other important and potential groups that are observed in polluted environments. Thus, Chloroflexi was found in sites with the application of activated sludge from the wastewater treatment industry^33^, and Anaerolinea was found in sites contaminated with petroleum^34^.

Several studies have shown the positive effect of applying industrial and municipal sludge in the improvement of soil fertility and the organic matter content^5,35,36^. The application of composted sludge, such as CTS, has promoted an increase in the growth of ornamental species^37^, cowpea and maize^38^. It suggests that composted tannery sludge could be recommended and used as fertilizer in agricultural soils, but it is necessary to decrease its high concentrations of Cr.

## Conclusion

There was a shift in the bacterial community over time with the application of composted tannery sludge. Interestingly, our study has shown that the less abundant bacterial groups were more influenced by the CTS than the most abundant bacterial groups. These bacterial groups were driven by the soil chemical properties, primarily Cr and the soil pH. In addition, the rhizosphere of plants demonstrated different effects on the bacterial community in soil with the application of the composted tannery sludge.

## Methods

The experiment with the CTS was conducted at the Agricultural Science Centre from the Federal University of Piauí, Brazil. The soil of the area is classified as a Fluvisol, and the upper 20 cm layer contains 10% of clay, 28% of silt, and 62% of sand. The compost was produced by mixing tannery sludge with sugarcane straw and cattle manure (volume ratio 1:3:1), and the composting process was performed over a 3-month period. The properties of the CTS were measured in the laboratory (Table 1). The water content was determined after drying the samples at 105 °C in an oven for 24 h; the pH was directly read, and the total solids were measured by drying the samples at 65 °C. The total organic C content was evaluated using dichromate oxidation of the samples under external heating. The total N content was determined using the Kjeldahl method after sulphuric acid digestion of the samples. The total Ca, Mg, K, P, S, Na, Zn, Cu, Cd, Pb, Ni, and Cr concentrations were determined by atomic absorption spectrophotometry after nitric acid digestion of the samples in a microwave oven^39^.

**Figure 6:**
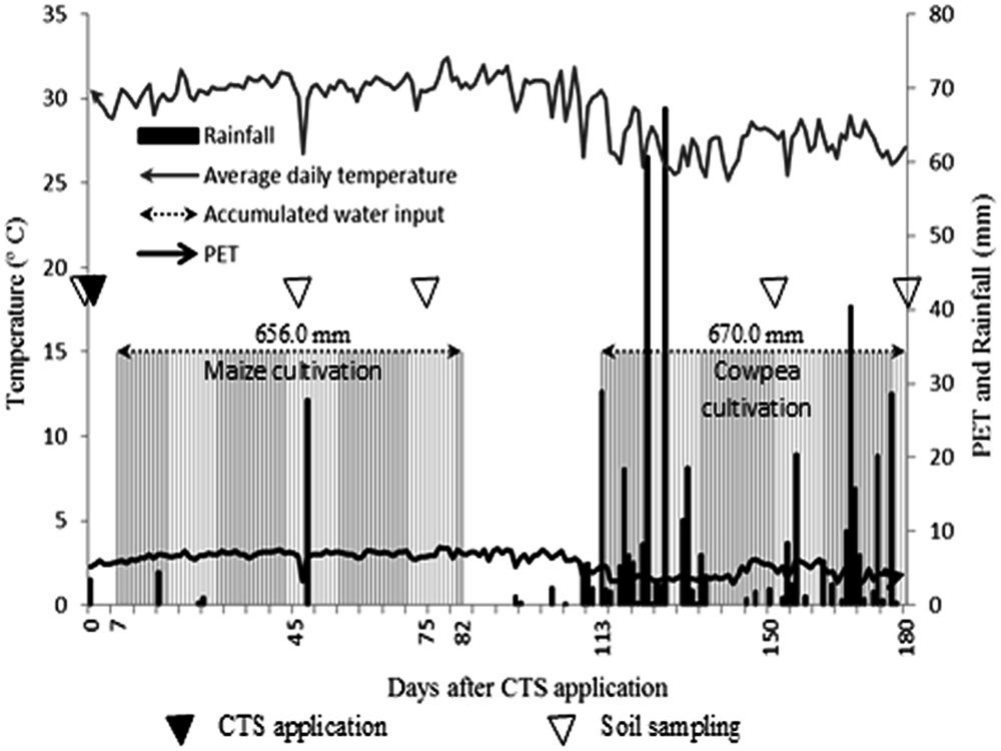
Timeline of the experiment (climatic data, CTS application, soil sampling, maize and cowpea cultivation, input of water). PET - potential evapotranspiration.

We evaluated the application of CTS at five rates: 0 (control), 2.5, 5, 10, and 20 t/ha of CTS (dry basis). The CTS treatments were applied to plots of 20 m^2^ (four replicates) by spreading it on the soil surface and incorporating it into the 20-cm layer with a harrow. In this experiment, we selected maize and cowpea, because they are the primary crops in our state. The CTS was applied 10 days before maize (*Zea mays* L.) AG 1051 was sown, and the plants were grown at a density of 5 plants m^−1^ (approximately 62,000 plants ha^−1^) for 75 days. After this period, cowpea [*Vigna unguiculata* (L.) Walp.], cv. BRS Tumucumaque, was sown at a density of 6 plants m^−1^ (approximately 120,000 plants ha^−1^) for 68 days. The plants were grown using natural rain as water. The timeline of the experiment is shown in Fig. 6.

To measure the effect of the CTS over time on the bacterial community, soil samples were collected from each plot at 0, 45, and 75 days (during the maize growth) and 150 and 180 days (during the cowpea growth) after the CTS application (from January to June 2015). Four soil samples were collected in each plot (0–20 cm), sieved (2 mm), and stored at −20 °C prior to analysis.

Soil DNA was extracted from 0.5 g (total humid weight) of the soil using a Power Lyzer Power Soil DNA Isolation Kit (MoBIO Laboratories, Carlsbad, CA, USA) according to the manufacturer’s instructions. The DNA extraction was performed in triplicate for each soil sample. The quality and concentration of the extracted DNA was determined using a NanoDrop 2000 spectrophotometer (Thermo Scientific, Waltham, USA).

The V4 region of the 16S rRNA gene was amplified using region-specific primers (515 F/806 R)^40^. Each 25 μL Polymerase Chain Reaction (PCR) reaction volume contained the following: 12.25 μL of nuclease-free water (Certified Nuclease-free, Promega, Madison, WI, USA), 5x Buffer, 2 mM MgCl_2_, 10 mM dNTPs, primers (40 μM 515 YF and 10 μM 806 R), 1.0 unit of Platinum Taq polymerase High Fidelity at a concentration of 0.5 μL (Invitrogen, Carlsbad, CA, USA), and 2.0 μL of template DNA. In addition, a control reaction was performed using water instead of DNA. The conditions for the PCR were as follows: 95 °C for 3 min to denature the DNA, with 35 cycles at 98 °C for 20 s, 55 °C for 20 s, and 72 °C for 30 s, with a final extension of 3 min at 72 °C to ensure complete elongation.

After indexing, the PCR products were purified using Agencourt AMPure XP – PCR purification beads (Beckman Coulter, Brea, CA, USA) according to the manufacturer’s instructions and quantified using a dsDNA BR assay Kit (Invitrogen, Carlsbad, CA, USA) on a Qubit 2.0 fluorometer (Invitrogen, Carlsbad, CA, USA). Once quantified, an equimolar concentration of each library was pooled into a single tube. After quantification, the molarity of the pool was determined and diluted to 2 nM, denatured, and then diluted to a final concentration of 8.0 pM using a 20% PhiX (Illumina, San Diego, CA, USA) spike to load into an Illumina MiSeq sequencing machine (Illumina, San Diego, CA, USA).

Sequence data were processed using QIIME following the UPARSE standard pipeline according to Brazilian Microbiome Project ((http://www.brmicrobiome.org/#!16s-profiling-pipeline-illumina/czxl)^41^, producing an OTU table and a set of representative sequences. Briefly, the reads were truncated at 240 bp and quality-filtered using a maximum expected error value of 0.5. Pre-filtered reads were dereplicated and singletons were removed and filtered for additional chimeras using the RDP gold database using USEARCH 7.0. These sequences were clustered into OTUs at a 97% similarity cut-off following the UPARSE pipeline. After clustering, the sequences were aligned and taxonomically classified against the Greengenes database (version 13.8).

Prior to the statistical analyses, the physicochemical parameters (except pH) and the OTU’s data sets were ln (Ax + 1) transformed, where x stands for the parameter value or abundance value and Ax makes 2 by taking the lowest value higher than zero for x. It was done in order to down-weigh high abundance values and approximates a normal distribution of the data (for rationale see^42^).

No observed effect concentrations (NOECs) were calculated for all physicochemical parameters and OTU’s separately (Table S1). Effects were considered to be consistent when they showed statistically significant deviations pointing in the same direction for at least two consecutive sampling days. The NOEC calculations were performed by using the Williams test^43^, which assumes a monotonic increasing effect with increasing exposure dose. The Williams tests were performed with the Community Analysis computer program, version 4.3.05^44^, using a significance level of 0.05.

The physicochemical and OTUs data sets at level phyla and class were analysed by the principal response curve (PRC) method^45^ to show and test temporal changes in bacterial community and chemical properties of the soil caused by different rates of CTS as compared with those in the control (CTS-free soil) and also to quantify the contribution of each property to separate the treatments from the control. The PRC method is a multivariate technique which is based on the redundancy analysis ordination technique and was performed using the CANOCO Software package, version 5^46^.

The PRC analysis results in a diagram showing time on the x-axis and the first principal component of the treatment effects on the y-axis, herewith showing the difference in the composition of the microbial and chemical properties of the treatments compared to the controls as they develop over time^41^. The overall significance of the tannery sludge treatment regime on the variation in species composition (p ≤ 0.05) was tested by performing 999 Monte Carlo permutations^41^. The NOECs of the tannery sludge treatment per sampling date was calculated by applying the Williams test to the sample scores of the first principal component of each sampling date (for rationale see^44,47^).

Lastly to show the correlations between the treatment, time, physico-chemical parameters and the OTU’s, an RDA was performed for the OTU’s at the phyla and class level separately using the OTU’s as response variables, physico-chemical parameters as explanatory variables and time and treatment level as supplementary environmental variables. Only physico-chemicals explaining a significant part of the variation in OTU’s between the samples were included. This was tested by performing Monte Carlo permutation tests (999 permutations) including the single physico-chemical parameters as explanatory variable, sampling date as covariable and by permuting within covariables only.

## Electronic supplementary material

Fig. S1

Table S1
