# Supplementary material for: Less abundant bacterial groups are more affected than the most abundant groups in composted tannery sludge-treated soil
Source: Sci Rep. 2018 Aug 6;8:11755. doi: 10.1038/s41598-018-30292-1 (PMC6079073; doi:10.1038/s41598-018-30292-1)
Supplement: Supplementary file 1 — Fig. S1 [file 41598_2018_30292_MOESM1_ESM.pdf]

Less abundant bacterial groups are more affected than the most abundant groups in composted tannery sludge-treated soil

Ana Roberta Lima Miranda, Jadson Emanuel Lopes Antunes, Fabio Fernando de Araujo, Vania Maria Maciel Melo, Walderly Melgaco Bezerra, Paul J. Van den Brink, and Ademir Sergio Ferreira de Araujo

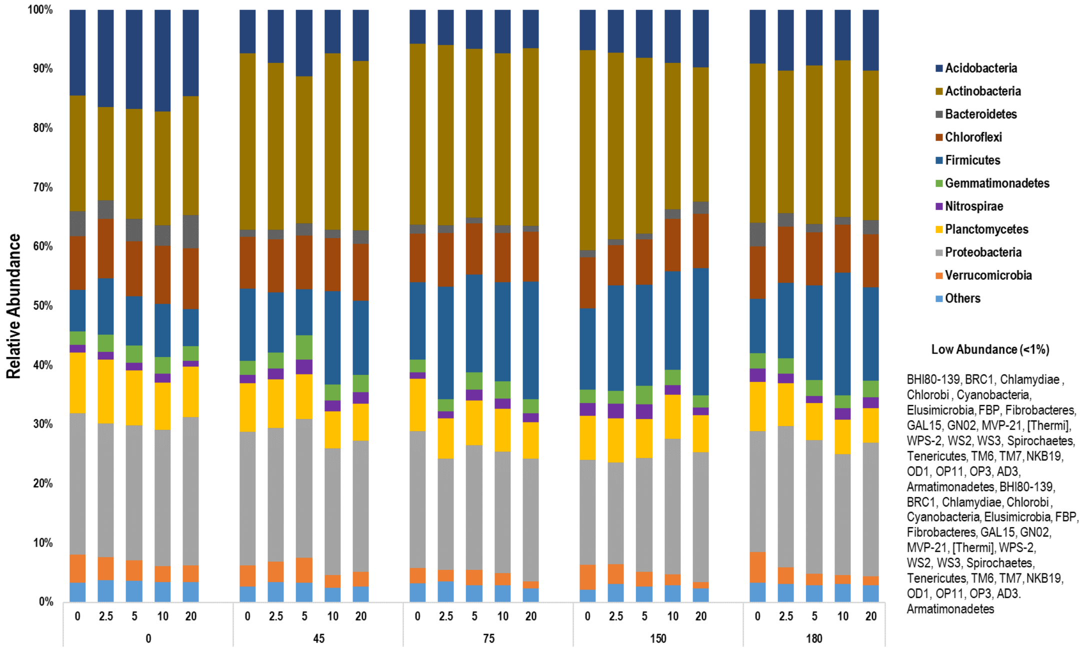

Fig. S1. Taxonomy at the Phyla level above 1% of relative abundance for all time and treatments after eight years application of the composted tannery sludge (CTS). The numbers 0, 45, 75, 150 and 180 are time sampling (days) after application of the CTS and the numbers 0, 2.5, 5, 10 and 20 are rates of the CTS (Mg ha^{-1}).
