# Supplementary material for: Less abundant bacterial groups are more affected than the most abundant groups in composted tannery sludge-treated soil
Source: Sci Rep. 2018 Aug 6;8:11755. doi: 10.1038/s41598-018-30292-1 (PMC6079073; doi:10.1038/s41598-018-30292-1)
Supplement: Supplementary file 2 — Table S1 [file 41598_2018_30292_MOESM2_ESM.pdf]

Less abundant bacterial groups are more affected than the most abundant groups in composted tannery sludge-treated soil

Ana Roberta Lima Miranda, Jadson Emanuel Lopes Antunes, Fabio Fernando de Araujo, Vania Maria Maciel Melo, Walderly Melgaco Bezerra, Paul J. Van den Brink, and Ademir Sergio Ferreira de Araujo

Table S1. OTU's and physico-chemical parameters showing consistent (two consecutive sampling dates) NOEC values. NOEC values are provided in ton/ha and the sign indicates the direction of the effect.

Data set	Days				
	**0**	**45**	**75**	**150**	**180**
**Bacterial phyla**					
All OTU's (PCA)	10	2.5	2.5	5	> 20
Acidobacteria	> 20	> 20	5+	2.5+	> 20
Chlamydiae	> 20	> 20	10-	10-	10-
Fibrobacteres	> 20	2.5+	10+	> 20	0+
OP3	> 20	0+	2.5+	5+	> 20
Planctomycetes	5-	5-	0-	> 20	2.5-
Verrucomicrobia	5-	> 20	10-	2.5-	0-
WS3	5+	2.5+	2.5+	5+	> 20
**Bacterial classes**					
All OTU's (PCA)	10	2.5	> 20	2.5	> 20
JG37-AG-4	0-	5-	> 20	5-	> 20
Acidobacteria-5	5-	5-	> 20	5-	2.5-
Acidobacteria-6	5+	> 20	2.5+	2.5+	> 20
RB25	> 20	> 20	0+	2.5+	0+
Solibacteres	10-	5-	2.5-	10-	5-
Sva0725	> 20	0+	> 20	0+	0+
[Chloracidobacteria]	> 20	2.5+	> 20	5+	10+
iii1-8	> 20	> 20	5+	0+	> 20
MB-A2-108	> 20	2.5+	0+	> 20	> 20
Nitriliruptoria	2.5+	2.5+	10+	2.5+	10+
Rubrobacteria	5+	10+	2.5+	10+	2.5+
At12OctB3	0+	2.5+	> 20	> 20	> 20
Cytophagia	10+	10+	> 20	5+	> 20
Chlamydiia	> 20	> 20	10-	10-	10-
BSV26	10-	10-	> 20	> 20	> 20
Anaerolineae	10+	10+	> 20	5+	0+
C0119	> 20	10-	> 20	10-	5-
Ktedonobacteria	10-	2.5-	2.5-	> 20	> 20
S085	0+	2.5+	2.5+	2.5+	> 20
TK17	10+	2.5+	> 20	5+	> 20
4C0d-2	5-	5-	> 20	> 20	> 20
Fibrobacteria	> 20	2.5+	10+	> 20	0+
Clostridia	> 20	> 20	10+	5+	2.5+
Gemm-2	5+	0+	> 20	10+	2.5+
Gemm-5	0+	2.5+	> 20	> 20	> 20
koll11	> 20	0+	5+	> 20	> 20
BD7-11	> 20	10-	10-	> 20	2.5-
OM190	10+	2.5+	> 20	0+	> 20
Phycisphaerae	5-	5-	0-	0-	5-
Planctomycetia	> 20	5-	10-	> 20	> 20

Deltaproteobacteria	10-	0-	0-	> 20	> 20
Gammaproteobacteria	10+	> 20	> 20	5+	0+
[Pedosphaerae]	> 20	5-	10-	5-	5-
[Spartobacteria]	5-	> 20	10-	2.5-	0-
PRR-12	5+	2.5+	2.5+	5+	> 20
**Chemical parameters**					
pH	0+	2.5+	2.5+	0+	0+
EC	10+	0+	10+	> 20	> 20
TOC	2.5+	2.5+	2.5+	10+	2.5+
P	5+	5+	10+	5+	2.5+
Ca	2.5+	0+	0+	2.5+	2.5+
Mg	2.5+	2.5+	> 20	0+	2.5+
Na	> 20	5+	> 20	0+	0+
Cr	0+	0+	0+	0+	0+

EC – Electric conductivity; TOC – total organic C
